# Vascular calcification on plain radiographs is associated with carotid intima media thickness, malnutrition and cardiovascular events in dialysis patients: a prospective observational study

**DOI:** 10.1186/1471-2369-14-27

**Published:** 2013-01-29

**Authors:** Won Suk An, Young Ki Son

**Affiliations:** 1 Department of Internal Medicine, Dong-A University, 3Ga-1, Dongdaesin-Dong, Seo-Gu, Busan 602-715, Republic of Korea; 2 Institute of Medical Science, Dong-A University College of Medicine, Busan, Korea

**Keywords:** Cardiovascular disease, Carotid intima media thickness, Malnutrition, Vascular calcification

## Abstract

**Background:**

Vascular calcification (VC) and carotid intima media thickness (CIMT) are strongly associated with cardiovascular (CV) disease. We hypothesized that significant VC on plain radiographs is associated with CIMT and CV events in dialysis patients. In addition, we evaluated risk factors for VC progression on plain radiographs in dialysis patients.

**Methods:**

In this 2-year observational, prospective study, 67 dialysis patients were included. We checked plain radiographs at baseline and after 2 years. Laboratory tests and malnutrition score were obtained at baseline, after 12 months, and after 24 months.

**Results:**

The mean age of patients was 56.3 ± 10.3 years and duration of dialysis was 41.3 ± 34.5 months. The prevalence of significant VC was 61.2% and the prevalence of carotid artery atheromatous plaques was 55.6%. Mean CIMT, malnutrition scores, CRP level and prevalence of carotid atheromatous plaques were significantly higher in patients with significant VC. Serum albumin and total iron binding capacity were significantly lower in patients with significant VC compared to patients without significant VC. During a mean observational period of 22 months, patients without significant VC showed lower CV events by the Kaplan-Meyer method (p = 0.010). Progression of VC was found in 35.7% among 56 patients followed up. Hemoglobin after 24 months was an independent factor for progression of VC (Exp(B) = 0.344, 95% Confidence Interval = 0.13 – 0.96, p = 0.034).

**Conclusions:**

Significant VC on plain radiograph was associated with CIMT, malnutrition, inflammation, and CV events in dialysis patients. Conditions which increase hemoglobin level may retard progression of VC in dialysis patients.

## Background

Cardiovascular disease is a major cause of morbidity and mortality in dialysis patients [1]. Vascular calcification (VC) is highly correlated with cardiovascular disease (CVD) and frequently detected in patients with chronic kidney disease (CKD) [2]. VC scores ≥ 3 on plain radiographs of the pelvis and hands are associated with coronary artery disease (CAD) in hemodialysis (HD) patients [3]. It was found that an abdominal aortic calcification (AAC) score of greater than 5 is related with the risk of CVD in dialysis patients [4]. The presence of medial artery calcification on plain radiography of the feet is also associated with prevalence of CAD and peripheral artery disease (PAD) in HD patients [5,6]. Therefore, these significant VCs on plain radiographs can be a vital source of information for CVD and cardiovascular mortality in dialysis patients.

Myocardial infarction and stroke were increased by the 0.1 mm increase of carotid intima media thickness (CIMT) in the meta-analysis [7]. Thoracic aortic calcification detected by computed tomography is associated with presence and severity of CIMT in the multi-ethnic study [8]. CIMT was greater in HD patients compared to the control group [9]. The presence of carotid plaques is also important risk factor for CVD and is common in HD patients compared to general population [10,11]. Valvular calcification has shown an association with inflammation, carotid atherosclerosis and arterial calcification in end-stage renal disease [12]. However, there is no previous study about the relationship between VC on plain radiograph and carotid intima media thickness (CIMT). Malnutrition is another important factor related to CVD and mortality in dialysis patients. In addition, malnutrition was related to valvular calcification and low fetuin-A levels which are marker of VC inhibition [13]. Still, there is no report about the association between VC on plain radiographs and malnutrition. VC on plain radiographs will be a useful clinical tool if those relationships are proven. Therefore, we hypothesized that significant VC on plain radiographs is associated with CIMT, malnutrition and cardiovascular events for two-year observational periods in dialysis patients. In addition, we evaluated risk factors including malnutrition for VC progression on plain radiographs in dialysis patients.

## Methods

### Patients

In this 24 month prospective observational study, we included dialysis patients aged 20 to 80 years and treated with dialysis for 6 or more months at Dong-A University dialysis center. Patients with a history of hospital admission due to CVD within 3 months, history of active infection within 3 months, malignancy and liver cirrhosis with Child class B or C were excluded. Finally we recruited 67 dialysis patients (41 HD patients and 26 peritoneal dialysis (PD) patients, 49.3% diabetes) who want to participate. Informed consent was obtained from all enrolled patients, and the study was approved by the Dong-A University Hospital Institutional Review Board. The enrolled HD patients received regular HD three times weekly using bicarbonate-based dialysate and polysulfone dialyzers (Fresenius, Bad Homburg, Germany). The enrolled PD patients received 4 exchanges per day using a standard regimen (8 L/day). All enrolled patients were interviewed and underwent a clinical examination at the initiation of the study. Their medical history and outpatient records were also evaluated in detail. We defined cardiovascular events as any one finding among the following findings: ischemic heart disease that needs treatment; sudden cardiac death; acute heart failure; PAD; hemorrhagic stroke; ischemic stroke.

### Vascular calcification

We checked the plain radiographs of the feet, hands, pelvis, and lateral lumbar spine and estimated the VC scores with previously reported methods at baseline and after 24 months. The abdominal aortic calcification (AAC) was graded using a previously reported system in which the location and severity of calcification deposits at each lumbar vertebral segment (Lumbar 1–Lumbar 4) were evaluated [4]. It has been reported that an AAC ≥ 5 increases the risk of CVD. VC scores of the pelvis and hands were evaluated in plain radiographic films. The radiographic films of the pelvis and hands were divided into 4 sections by two imaginary lines. The final score was the sum of all sections, ranging from 0 to 8. In a previous study, receiver operating characteristic curve analysis identified a VC score ≥ 3 as the best cut-off value associated with cardiovascular motility and cardiovascular events [3]. On radiographs of the feet, the score is 0 when medial artery calcification on plain radiographs of feet is absent, and the score is 1 when VC is present. Each VC is a useful predictor for CVD. We checked several plain radiographs and evaluated several VC sites on plain radiographs. And we defined significant VC as any one finding among the following findings on plain radiographs: AAC score ≥ 5; VC score of the hands and pelvis ≥ 3; medial artery calcification of the feet. We defined VC progression on plain radiographs as any one finding among the following findings on plain radiographs: increases of AAC score ≥3; increases of VC score of the hands and pelvis ≥1; newly developed medial artery calcification of the feet. AXIOM Aristors MX/VX (SIEMENS, Erlangen, Germany) radiographic equipment with a digital imaging system was used (exposure conditions, 45–50 kVp [4mAs]). Two nephrologists individually decided VC scores on plain radiographs without information from patients. Consensus was reached on the interpretation of all radiographs.

### Assessment of CIMT

Carotid Doppler ultrasonography was performed by a single operator and CIMT was measured; the operator was blinded about the history and laboratory findings of the patients. CIMT was defined as the distance between the leading edge of the first bright line and the leading edge of the second bright line containing intima-media interface and media-adventitia interface on the posterior wall of the both common carotid arteries (CCA). Measurements were taken over a 10-mm length using a B-mode high-resolution ultrasonography system (Philips Sonos 5500, Andover, MA, USA). When an atherosclerotic plaque was present at the measurement site, it was included in the measurement of the CIMT. Atherosclerotic plaques were defined as a protrusion into the lumen adding 50% to the thickness of the surrounding intima-media or maximal thickness of 1.5 mm in the both CCA. We used the maximal thickness of the intima-media complex as CIMT in this study.

### Laboratory measurements

Laboratory tests including hemoglobin (Hb), calcium, phosphorous, intact parathyroid hormone, serum albumin, total iron-binding capacity (TIBC), alkaline phosphatase, C-reactive protein (CRP), total cholesterol, triglycerides, low-density lipoprotein-cholesterol, high-density lipoprotein cholesterol (HDL), blood urea nitrogen and creatinine were obtained using fasting blood samplings. HbA1c was determined in diabetic patients. Blood samples and body mass index (BMI) were taken before HD and before Carotid Doppler ultrasonography in PD patients.

### Assessment of malnutrition

We used Comprehensive Malnutrition Inflammation Scores (MIS) to assess the status of malnutrition [14]. Seven components of Dialysis Malnutrition Score were revised to comprehensive MIS by adding three new items: BMI, serum albumin level, and TIBC [15]. The other 7 components of Comprehensive MIS are dry weight, dietary intakes, gastrointestinal symptoms, functional capacity, comorbidity including number of years on dialysis, loss of subcutaneous fat and muscle wasting. Comprehensive MIS has 10 components, each with four levels of severity, from 0 (normal) to 3 (severely abnormal). The sum of all 10 comprehensive MIS components ranges from 0 (normal) to 30 (severely malnourished); a higher score reflects a more severe degree of malnutrition and inflammation.

We evaluated body fat composition by using bioelectrical impedance analysis at baseline and after 24 months.

### Statistical analysis

We initially decided on a minimum sample size of 80 because the minimum correlation coefficient was 0.22 between VC on plain radiographs and CAD, based on our previous study. Therefore, we observed and evaluated cardiovascular events including CAD because we recruited a total of 67 dialysis patients in this study. All data are presented as the means ± SD. The non-parametric Mann–Whitney U test was used to compare baseline data according to significant VC, each VC scoring method and progression of VC on plain radiographs. The difference in frequency was tested using Pearson’s chi-square analysis. Spearman analysis was used for correlation analysis. The non-parametric Wilcoxon exact rank sum test was used to compare baseline data to 12 months data, and 24 months data. The non-parametric Wilcoxon exact rank sum test was also used to compare 12 months data to 24 months data. Cardiovascular events, coronary events and cardiovascular deaths according to significant VC and each VC scoring method were analyzed by Kaplan-Meier models using the log-rank test. Univariate and mulvariate logistic regression analysis was applied to determine the factors independently associated with progression of VC on plain radiographs. The adjusted variables included age, diabetes, albumin at 24 months, CRP at 24 months and Hb at 24 months. We rejected null hypotheses of no difference if p-values were less than 0.05. All statistical calculations were performed with SPSS software (version 19.0; SPSS Inc., Chicago, IL, USA).

## Results

### Clinical and laboratory findings according to significant VC

The mean age of enrolled dialysis patients was 56.3 ± 10.3 years and the duration of dialysis was 41.3 ± 34.5 months. The prevalence of significant VC was 61.2% and the prevalence of carotid artery atherosclerotic plaques was 55.6%. The baseline clinical findings of patients according to significant VC on plain radiographs are listed in Table 1. Those with significant VC were older, and had higher prevalence of diabetes. Mean CIMT (right: p = 0.006, left: p = 0.001), MIS (p = 0.007), CRP levels and prevalence of carotid artery atherosclerotic plaques (p = 0.003) were significantly higher in patients with significant VC compared to patients without significant VC (Table 1). Serum albumin and total iron binding capacity were significantly lower in patients in patients with significant VC compared to patients without significant VC. There were no significant differences of body fat composition according to significant VC. Mean levels of intact parathyroid hormone, calcium, and phosphorous were not significantly different in patients with and without significant VC. Left CIMT (r = 0.466, p = 0.011), right CIMT (r = 0.378, p = 0.043), MIS (r = 0.340, p = 0.006) and prevalence of atheromatous plaques (r = 0.424, p = 0.024) were significantly correlated with significant VC.

**Table 1:** Characteristics of study population according to significant vascular calcification

**Characteristics**	**Significant vascular calcification**		
	**Positive (n = 41)**	**Negative (n = 26)**	**P-value**
Age (years)	59.6 ± 9.2	51.12 ± 10.0	0.001
Male, n (%)	13(38.2%)	9(40.9%)	1.000
Diabetes, n (%)	26(75.8%)	8(24.2%)	0.015
HD/PD, n	26/15	15/11	0.415
CIMT, Right. (mm)	0.97 ± 0.19	0.77 ± 0.21	0.006
CIMT, Left (mm)	0.97 ± 0.21	0.74 ± 0.13	0.001
Carotid atheroma (%)	73.9	23.1	0.003
MIS	4.6 ± 2.7	3.3 ± 52.7	0.007
Dialysis duration (months)	44.6 ± 32.9	36.1 ± 37.1	0.333
Body mass index (kg/m^2^)	23.6 ± 3.0	23.1 ± 3.1	0.674
Hemoglobin (g/dL)	10.4 ± 1.0	10.5 ± 1.2	0.867
PTH (pg/mL)	295.5 ± 264.8	411.9 ± 343.3	0.158
Calcium (mg/dL)	8.8 ± 0.7	8.7 ± 0.7	0.811
Phosphorous (mg/dL)	4.7 ± 1.5	4.8 ± 1.5	0.719
Creatinine (mg/dL)	12.5 ± 16.2	10.3 ± 2.8	0.857
Albumin (g/dL)	3.9 ± 0.3	4.0 ± 0.3	0.035
TIBC (ug/dl)	245.0 ± 46.7	288.6 ± 33.7	0.017
CRP (mg/dL)	0.9 ± 1.5	0.2 ± 0.4	0.000

Abbreviations: *HD*, hemodialysis; *PD*, peritoneal dialysis; *CIMT*, carotid intima-media thickness; *MIS*, malnutrition-inflammation score; *PTH*, parathyroid hormone; *TIBC*, total iron binding capacity; *CRP*, C-reactive protein.

### Clinical and laboratory findings according to each VC scoring method

The prevalence of diabetes, age, both CIMT and CRP were significantly higher in patients with medial artery calcification of the feet than patients without medial artery calcification of the feet. TIBC and HDL cholesterol were significantly lower in patients with medial artery calcification of the feet than patients without medial artery calcification of the feet. There were no significant differences of prevalence of carotid artery atherosclerotic plaques, MIS and albumin between the groups with and without medial artery calcification of the feet (Table 2).

**Table 2:** Comparison of clinical and laboratory findings according to each VC scoring method on plain radiographs

	**Feet VC (+) (n = 29)**	**Feet VC (−) (n = 38)**	**Hands & Pelvis scores ≥3 (n = 27)**	**Hands & Pelvis scores <3 (n = 40)**	**AAC scores** ≥**5 (n = 22)**	**AAC scores < 5 (n = 45)**
Age (years)	60.0 ± 9.8*	53.4 ± 9.9	58.8 ± 1.0	54.6 ± 10.3	60.6 ± 7.4*	54.2 ± 11.0
DM (%)	21 (72.4)*	12 (31.6)	20 (74.1) *	13 (32.5)	13 (59.1)	20 (44.4)
CIMT, Right (mm)	1.0 ± 0.2*	0.8 ± 0.2	1.0 ± 0.2	0.8 ± 0.2	1.0 ± 0.2	0.8 ± 0.2
CIMT, Left (mm)	1.0 ± 0.3*	0.8 ± 0.2	1.0 ± 0.3*	0.8 ± 0.1	1.0 ± 0.3*	0.8 ± 0.2
Carotid atheroma (%)	73.3	42.9	86.7*	33.3	92.3*	34.8
MIS	4.6 ± 2.9	3.8 ± 2.6	4.4 ± 2.9	3.9 ± 2.7	5.0 ± 3.0*	3.7 ± 2.5
HDL (mg/dL)	37.5 ± 10.9*	43.3 ± 10.3	38.0 ± 11.4*	42.7 ± 10.3	40.8 ± 11.0	40.8 ± 11.0
CRP (mg/dL)	1.1 ± 1.8*	0.3 ± 0.4	0.8 ± 1.5	0.5 ± 1.1	0.8 ± 1.3*	0.5 ± 1.2
TIBC (ug/dl)	231.6 ± 41.3*	279.6 ± 41.0	247.7 ± 42.7	273.6 ± 46.9	253.3 ± 50.7	270.4 ± 44.1
Albumin (g/dL)	3.9 ± 0.3	4.0 ± 0.3	3.9 ± 0.3	3.9 ± 0.3	3.8 ± 0.3*	4.0 ± 0.3

Abbreviations: *VC*, vascular calcification; *DM*, diabtes mellitus; *CIMT*, carotid intima-media thickness; *MIS*, malnutrition-inflammation score; *HDL*, high density lipoprotein cholesterol; *TIBC*, total iron binding capacity; *CRP*, C-reactive protein.

*p < 0.05; significant different from patients with VC(−) or lower score in each group.

The prevalence of carotid artery atherosclerotic plaques, age and left CIMT were significantly higher in patients with hands and pelvis VC score ≥ 3 than patients with hands and pelvis VC score < 3. HDL was significantly lower in patients with hands and pelvis VC score ≥ 3 than patients with hands and pelvis VC score < 3.

The prevalence of carotid artery atherosclerotic plaques, age, left CIMT, MIS and CRP were significantly higher in patients with AAC ≥ 5 than patients with AAC < 5. Serum albumin was significantly lower in patients with AAC ≥ 5 than patients with AAC < 5. There were no significant differences of prevalence of diabetes, HDL cholesterol and TIBC between the groups according AAC ≥ 5 or < 5.

Nineteen (42.2%) of 45 patients with AAC scores < 5 had significant VC score on other parts, 14 of 40 patients (35.0%) with hand and pelvis VC score < 3 had significant VC score on other parts, and 12 (31.6%) of 38 patients without medial artery calcification of the feet had significant VC score on other parts.

### VC progression

**Figure 1:**
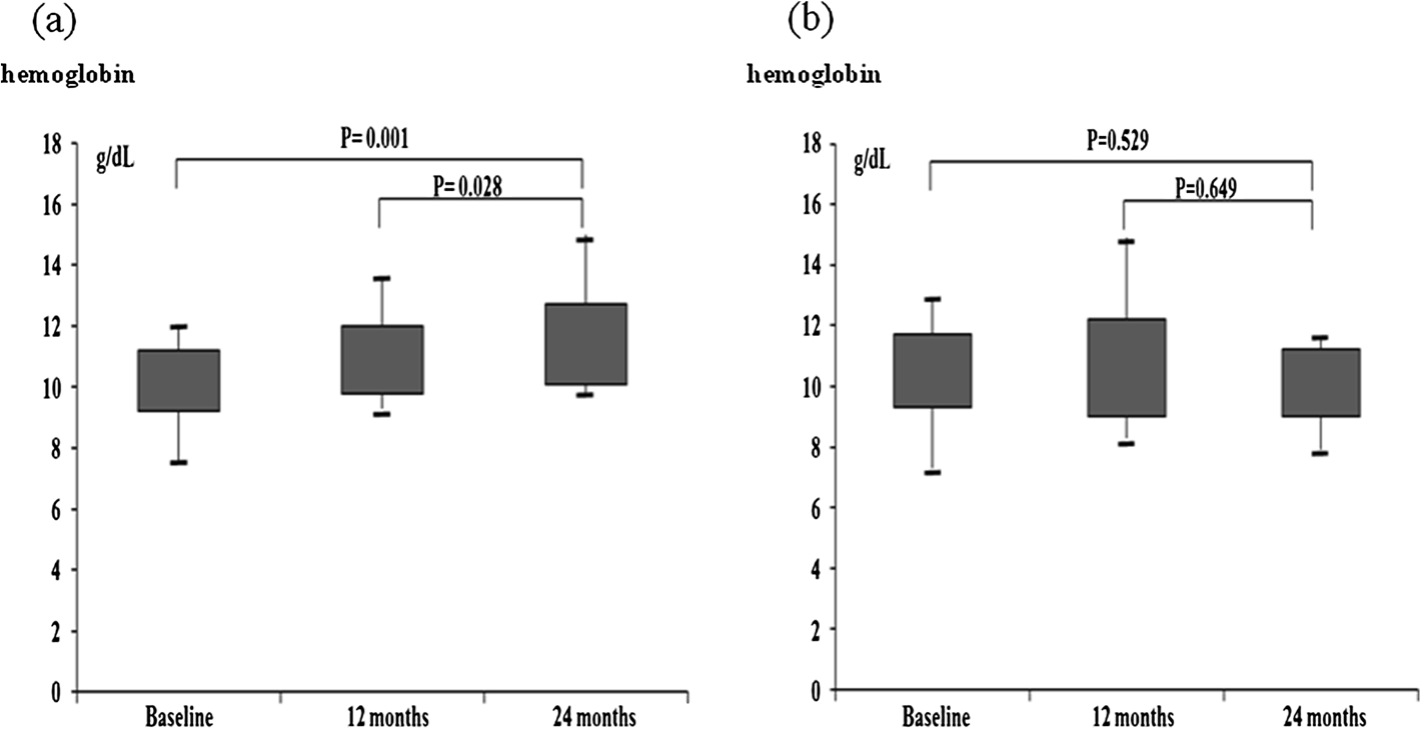
Hemoglobin levels according to elapsed time in patients showed vascular calcification non-progression (a) and vascular calcification progression (b) on plain radiographs.

VC progression on plain radiographs was found in 20 patients (35.7%, 47.6% of PD patients and 29.6% of HD patients) among 56 patients followed up. Increases of AAC score ≥3 in 12 patients, increases of VC score of hands & pelvis ≥1 in 3 patients and newly developed medial artery calcification of the feet in 2 patients were found. VC progressions based on two different VC scoring methods was also found in two patients. Three different VC progressions were even found in one patient. Fourteen of 20 patients (70%) who showed progression of VC throughout the study already had a significant VC at baseline and 18 of 36 patients (50%) who showed no progression of calcification patients had a significant VC at baseline. During follow-up period, Hb levels were significantly increased according to elapsed time in patients who did not show VC progression on plain radiographs (10.3 ± 1.0 g/dL at baseline, 10.9 ± 1.1 g/dL after 1 year, 11.4 ± 1.3 g/dL after 2 years) (Figure 1). Erythropoietin dose per month was not significantly higher in patients who showed VC progression on plain radiographs (Darbepoetin alpha: 282.9 ± 130.6 mcg/month, erythropoietin-beta: 49224.1 ± 39854.1 IU/month [patients with VC progression] vs. Darbepoetin alpha: 238.6 ± 210.5 mcg/month, erythropoietin-beta: 49980.2 ± 33146.0 IU/month, [patients without VC progression]). There was no significant difference of iron replacement in the enrolled dialysis patients (3701.5 ± 2017.4 mg/week [patients with VC progression] vs. 3251.7 ± 1027.4 mg/week [patients without VC progression]). HDL (41.3 ± 11.6 mg/dL vs. 47.4 ± 13.9 mg/dL) and serum albumin levels (3.96 ± 0.27 g/dL vs. 4.14 ± 0.33 g/dL) were also significantly increased at the end of follow-up period compared to baseline in patients who did not show VC progression. Hb levels after 24 months were an independent factor for VC progression on plain radiographs (Exp(B) = 0.344, 95% Confidence Interval = 0.13 – 0.96, p = 0.034)(Table 3).

**Table 3:** Logistic regression analysis for progression of vascular calcification

	**Univariate**		**Multivariate**	
**Variable**	**Exp(B) (95% CI)**	**p value**	**Exp(B) (95% CI)**	**p value**
Age	1.023(0.97-1.08)	0.435	0.951(0.85-1.06)	0.375
Diabetes	0.584(0.19-1.76)	0.340	0.931(0.17-5.09)	0.934
Albumin at 24 months	0.175(0.03-1.24)	0.081	0.517(0.04-6.78)	0.615
CRP at 24 months	1.575(0.54-4.63)	0.409	3.640(0.20-65.31)	0.380
Hb at 24 months	0.308(0.11-0.88)	0.029	0.344(0.13-0.96)	0.034

Abbreviations: *CI*, Confidence Interval; *CRP*, C-reactive protein; *Hb*, Hemoglobin.

### Cardiovascular events according to VC

**Figure 2:**
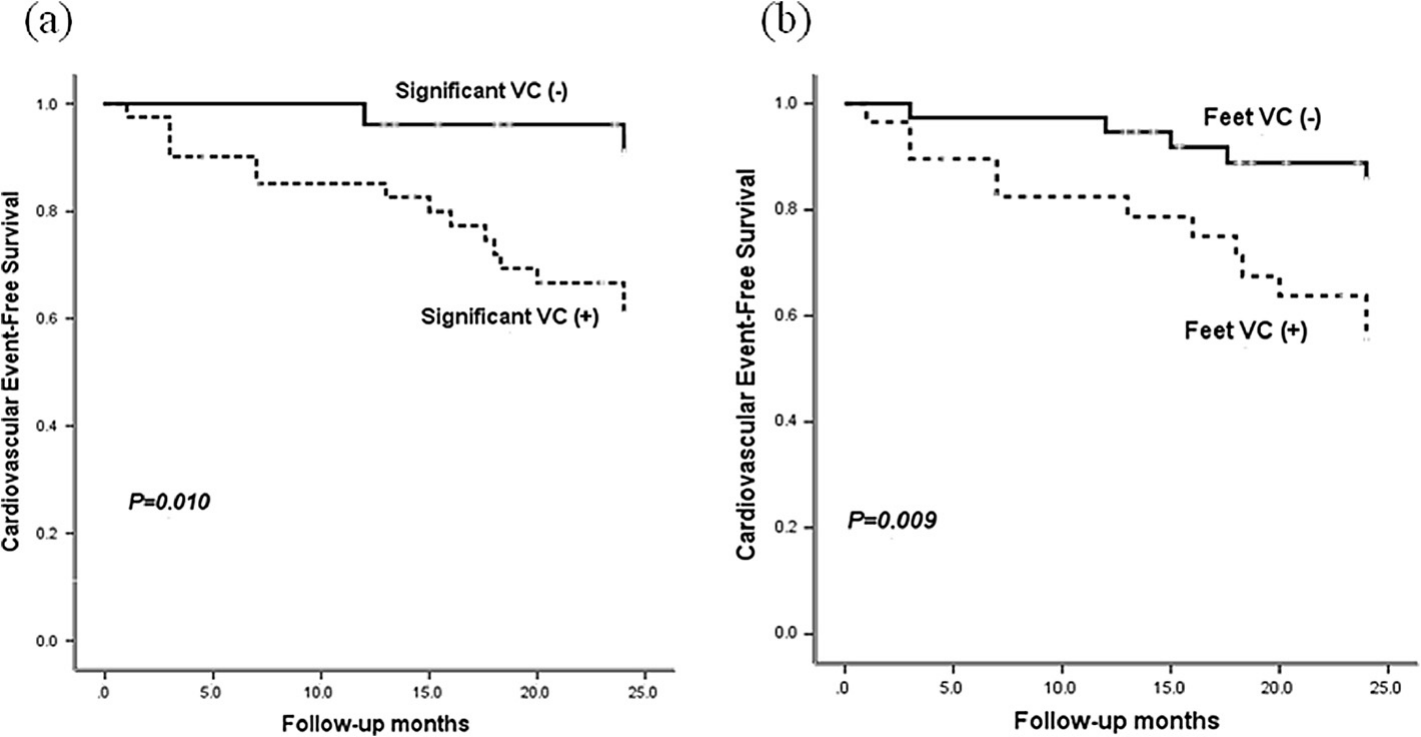
Cardiovascular events free survival according to significant vascular calcification (a), medial artery calcification of the feet (b).

**Figure 3:**
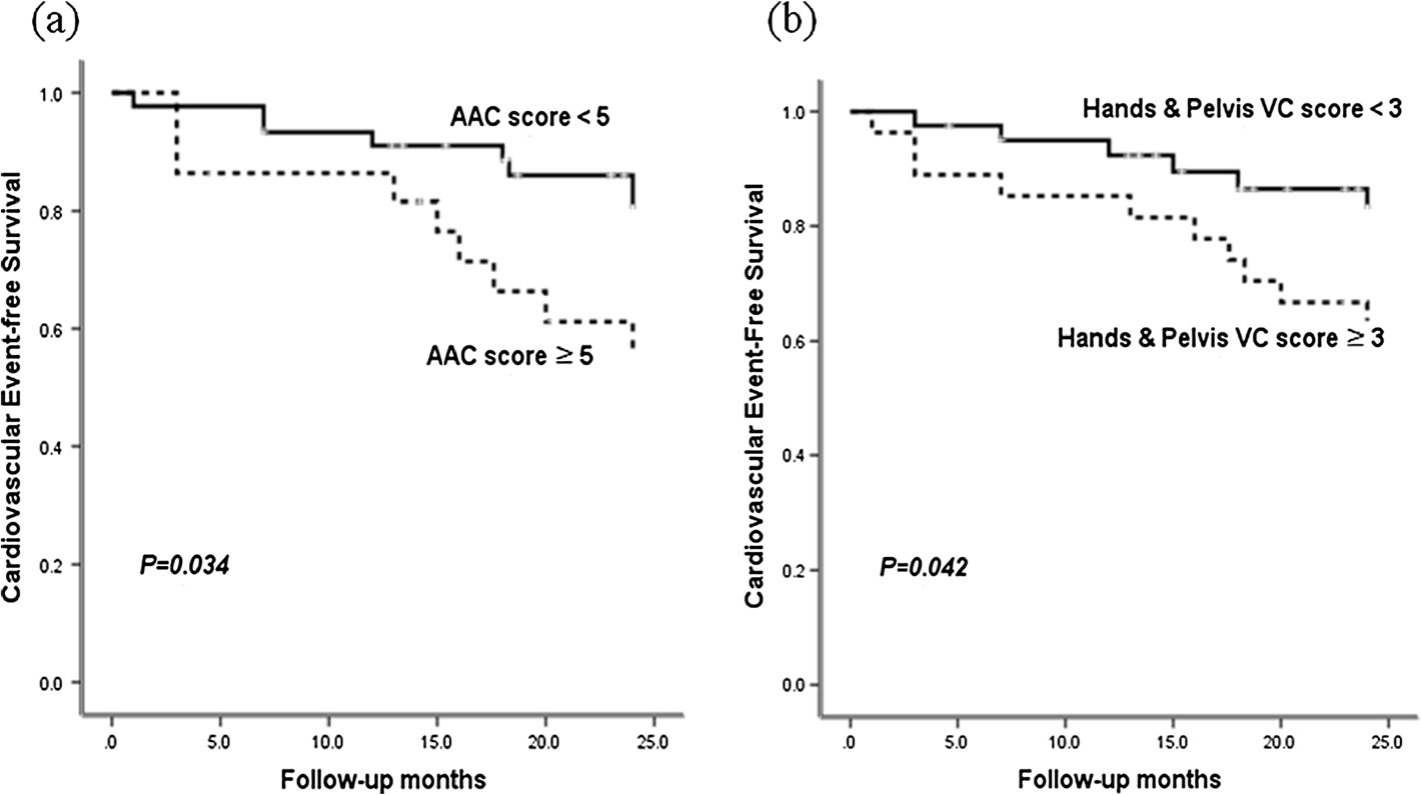
Cardiovascular events free survival according to AAC score ≥ 5 (a) and hands and pelvis VC score ≥ 3 (b).

During an observational period of 22 months, there were 6 cardiovascular deaths (three sudden cardiac deaths, two fatal myocardial infarctions and one intracerebral hemorrhage) among 8 deaths. There was no significant difference of cardiovascular mortality by significant VC on the plain radiographs. CAD was developed in 13 patients (19.4%) and 17 patients (25.4%) suffered from cardiovascular events. Significantly fewer cardiovascular events (p = 0.010) were observed in patients without significant VC compared to patients with significant VC (Figure 2). In each VC scoring method, significantly lower cumulative cardiovascular events were observed in patients without medial artery calcification of the feet (log rank = 6.8; p = 0.009), with hands and pelvis VC score < 3 (log rank = 4.1; p = 0.04) and with AAC score < 5 (log rank = 4.5; p = 0.034) compared to patients with medial artery calcification of the feet or with higher VC scores (Figures 2, 3). Coronary events were lower, but not significant, in patients without significant VC compared to patients with significant VC (p = 0.07). In each VC scoring method, coronary events were not significantly different in patients without medial artery calcification of the feet (p = 0.135), with hands and pelvis VC score < 3 (p = 0.340) and with AAC score < 5 (p = 0.097) compared to patients with medial artery calcification of the feet or with higher VC scores.

## Discussion

In this study, we observed that carotid intima media was thicker in patients with any one significant VC than in patients without significant VC. The same results were observed in patients who showed meaningful VC according to each VC scoring method. Furthermore, cardiovascular events were more frequent in patients with any one significant VC than patients without significant VC for the two-year observation period. It is of note that both significant VC defined by using three VC scoring methods and medial artery calcification by checking both feet AP view are more reliable predictors for cardiovascular events than any other single VC scoring method. We also found that VC evaluation on plain radiographs by single method overlooked over 30% of other significant VC sites in dialysis patients. These results suggest that we can explain cardiovascular risks to the patients not only with single VC scoring methods but also with any one significant VC defined by using several VC scoring methods. VC scoring methods on plain radiographs are less quantitative, but those are cheaper diagnostic methods than VC scores using computed tomography (CT). To our knowledge, this is the first observational study to compare clinical findings according to several VC scoring methods on plain radiographs and to prove the necessity of checking several VC sites for evaluation of possible CVD.

Vascular calcification and progression of aortic arch was associated with higher risk of mortality [16]. VC progression on the plain radiographs was found in the one third of patients followed for the short period of two years in our study. It is of note that VC of abdominal aortic area progressed in 60% of patients with VC progression, and VC of other sites also progressed in 40% of patients with VC progression. In addition, VC progression of multiple sites on the plain radiographs was found in 15% of patients with VC progression. Therefore, VC evaluation by using several plain radiographs is more useful than using single VC scoring method for close monitoring of VC progression. VC at baseline has proven to be one of the most important factors for VC progression in several studies using CT [17-20]. Patients with a zero coronary artery calcification (CAC) score at baseline showed no progress to a CAC Score >30 over 18 months [18]. However, 30% of patients who showed VC progression had no significant VC at baseline in our present study. Consistent with our results, 32.6% among patients with an initial calcification score of zero on CT of superficial femoral artery showed VC development in a previous study [19]. Based on these results, we can suggest that VC can easily progress if patients’ environments are continuously compatible with VC formation or changed to conditions for VC formation.

Chronic inflammation and oxidative stress prominent in uremic status are related to VC [21]. Older age, dyslipidemia and diabetes are also important factors affecting on VC formation [22]. Low HDL was associated with rapid progression of CAC determined by EBCT in HD patients [22]. In this present study, HDL was significantly lower in patients with medial artery calcification of the feet or hands and pelvis VC score ≥ 3 on the plain radiographs. In addition, HDL was significantly increased compared to baseline levels in patients who did not show VC progression. However, there was no difference of HDL according to significant calcification of abdominal aorta in this study. Based on these results, HDL is closely related with VC formation in small to medium vessels. The prevalence of diabetes was significantly higher in patients with medial artery calcification of feet and hands and pelvis VC score ≥ 3, but there was no difference of diabetes according to significant abdominal aortic calcification. These results partially support that different pathogenic mechanisms may be related to VC locations. Further studies are necessary to elucidate the pathogenesis on VC formation according to vessel size or location.

Anemia is associated with high mortality in patients who receive maintenance dialysis treatment and increased survival was associated with rising hemoglobin over a 2-year observation period [23,24]. Mortality can be also affected by erythropoietin responsiveness in HD patients [25]. In addition, hemoglobin reflects well several conditions including malnutrition in dialysis patients. We found increasing levels of hemoglobin over a 2-year observation period in patients with non-progression of VC, which was not related to the doses of supplemental iron or erythropoietin. This may indicate that conditions in favour of increase in hemoglobin level, such as lack of inflammation and oxidative stress, may be also supportive against VC. Albumin and MIS were associated with significant VC and AAC score ≥ 5 and increased albumin was associated with VC progression in the present study. These results suggest that nutritional status reflecting several conditions may affect VC. However, these conditions may be different with environment with high and adequate hemoglobin levels just by blood transfusion or high erythropoietin supplementations without correction of inflammation, oxidative stress and malnutrition. Further investigations are necessary to elucidate the effect of hemoglobin correction on preventing VC progression.

A broader interpretation of these study results in dialysis patients is limited by the small sample size and short observational period. Despite these limitations, we demonstrated that significant VC defined by using several VC scoring methods was useful tool for predicting CV events and it was related with CIMT and malnutrition.

## Conclusions

In conclusion, significant VC on plain radiograph was associated with CIMT, malnutrition, inflammation, and CV events in dialysis patients. Conditions which increase hemoglobin level may retard progression of VC on the plain radiograph in dialysis patients.

## Abbreviations

VC: Vascular calcification; CIMT: Carotid intima media thickness; CV: Cardiovascular; CVD: Cardiovascular disease; CKD: Chronic kidney disease; CAD: Coronary artery disease; HD: Hemodialysis; AAC: Abdominal aortic calcification; PAD: Peripheral artery disease; PD: Peritoneal dialysis; CCA: Common carotid arteries; Hb: Hemoglobin; TIBC: Total iron-binding capacity; CRP: C-reactive protein; HDL: High-density lipoprotein cholesterol; BMI: Body mass index; MIS: Malnutrition Inflammation Scores; CT: Computed tomography; CAC: Coronary artery calcification

## Competing interests

All authors declare that they have no competing interests.

## Authors’ contributions

WSA and YKS designed the research protocol and wrote the paper. WSA contributed to acquisition of data and analyzed the data. WSA and YKS were involved in revising the manuscript critically for important intellectual content and have given final approval of the version to be published. Both authors read and approved the final manuscript.

## Pre-publication history

The pre-publication history for this paper can be accessed here:

http://www.biomedcentral.com/1471-2369/14/27/prepub
